# Interventions for Adolescent Substance Abuse: An Overview of Systematic Reviews

**DOI:** 10.1016/j.jadohealth.2016.06.021

**Published:** 2016-10

**Authors:** Jai K. Das, Rehana A. Salam, Ahmed Arshad, Yaron Finkelstein, Zulfiqar A. Bhutta

**Affiliations:** a Division of Women and Child Health, Aga Khan University, Karachi, Pakistan; b Division of Emergency Medicine, The Hospital for Sick Children, Toronto, Ontario, Canada; c Division of Clinical Pharmacology and Toxicology, The Hospital for Sick Children, Toronto, Ontario, Canada; d Centre for Global Child Health, The Hospital for Sick Children, Toronto, Ontario, Canada; e Center of Excellence in Women and Child Health, The Aga Khan University, Karachi, Pakistan

**Keywords:** Adolescent health, Substance abuse, Drug abuse

## Abstract

Many unhealthy behaviors often begin during adolescence and represent major public health challenges. Substance abuse has a major impact on individuals, families, and communities, as its effects are cumulative, contributing to costly social, physical, and mental health problems. We conducted an overview of systematic reviews to evaluate the effectiveness of interventions to prevent substance abuse among adolescents. We report findings from a total of 46 systematic reviews focusing on interventions for smoking/tobacco use, alcohol use, drug use, and combined substance abuse. Our overview findings suggest that among smoking/tobacco interventions, school-based prevention programs and family-based intensive interventions typically addressing family functioning are effective in reducing smoking. Mass media campaigns are also effective given that these were of reasonable intensity over extensive periods of time. Among interventions for alcohol use, school-based alcohol prevention interventions have been associated with reduced frequency of drinking, while family-based interventions have a small but persistent effect on alcohol misuse among adolescents. For drug abuse, school-based interventions based on a combination of social competence and social influence approaches have shown protective effects against drugs and cannabis use. Among the interventions targeting combined substance abuse, school-based primary prevention programs are effective. Evidence from Internet-based interventions, policy initiatives, and incentives appears to be mixed and needs further research. Future research should focus on evaluating the effectiveness of specific interventions components with standardized intervention and outcome measures. Various delivery platforms, including digital platforms and policy initiative, have the potential to improve substance abuse outcomes among adolescents; however, these require further research.

Adolescence is recognized as the period for onset of behaviors and conditions that not only affect health limited to that time but also lead to adulthood disorders. Unhealthy behaviors such as smoking, drinking, and illicit drug use often begin during adolescence; they are closely related to increased morbidity and mortality and represent major public health challenges. Unemployment, poor health, accidents, suicide, mental illness, and decreased life expectancy all have drug misuse as a major common contributing factor [1], [2]. Substance abuse has a major impact on individuals, families, and communities as its effects are cumulative, contributing to costly social, physical, and mental health problems [3]. Several factors can enhance the risk for initiating or continuing substance abuse including socioeconomic status, quality of parenting, peer group influence, and biological/inherent predisposition toward drug addiction [4]. This culminates in a cycle where these individuals cease to perform as effective members of society and instead are consumed by their addictions [5].

Globally, tobacco use is the leading preventable cause of premature death and most adult smokers initiate smoking in adolescence [6], [7]. The prevalence of smoking in girls and boys varies across countries; 1 in every 10 girls aged 13–15 years and 1 in every 5 boys aged 13–15 years use tobacco [2], [6]. Smoking rates are generally highest in Europe and the Western Pacific regions while cigarette smoking is decreasing among younger adolescents in most high-income countries (HICs) and in some low- and middle-income countries. Approximately 4% of the global burden of disease is attributable to alcohol use [8]. Alcohol consumption among adolescents and young adults is increasing globally; however, it is decreasing in most HICs in Europe and North America [2], [9]. Currently, the World Health Organization (WHO) European Region and WHO Region of the Americas report the highest proportions of drinkers among adolescents while the WHO South-East Asia Region and WHO Eastern Mediterranean Region have the lowest [9]. In general, men drink more alcohol than women, but the sex difference is smaller at younger age. Cannabis use is associated with a decline in intelligence quotient scores before age 18 years and an increase in the risk of injury among adults. Unlike other substances, in many countries, boys and girls show similar prevalence of ever-using cannabis.

Efforts should be concerted on early identification, awareness and prevention programs, and routine monitoring of adolescent health data. Given the prevailing burden and impact of substance abuse in children and adolescents, it is essential that effective interventions and delivery platforms on enhancing social skills, problem-solving skills, and self-confidence are identified and implemented [10]. Standardized screening tools on identifying adolescents at high risk are available and outlined in the American Academy of Pediatrics and National Institute on Alcohol Abuse and Alcoholism publications [11], [12], [13], [14]. School-based surveys of adolescents monitor a number of these health-related behaviors among adolescents at the country level. The focus should be targeting modifiable risk factors and enhancing protective factors through family, school, and community prevention programs [15]. The various types of prevention programs can be delivered via school, community, and health care systems with general goals of case finding with accompanying referral and treatment or risk factor reduction [16], [17], [18].

This article is part of a series of reviews conducted to evaluate the effectiveness of potential interventions to improve adolescent health and well-being. We developed a conceptual framework based on existing conceptual frameworks [19], [20] and consultations and deliberations with the global experts in the field of adolescent health, and based on the recommendations, we identified a set of interventions to be incorporated in our review process. The interventions were chosen from the existing work on the basis of proven and potential effectiveness to improve adolescent health outcomes and access to primary health care and commodities for adolescents [20], [21], [22], [23]. Detailed conceptual framework, methodology, and other potential interventions have been discussed in separate articles [24], [25], [26], [27], [28], [29], [30]. Our conceptual framework depicts the individual and general risk factors through the life cycle perspective that can have implications at any stage. However, the focus of this overview is to evaluate potential interventions and delivery platforms targeting adolescent age group only and impact quality of life thereon [25]. We focused on risk factors including risky sexual behaviors, unintended pregnancies, violence, risky driving (including speeding and drunk driving), undernutrition, obesity, infections, and mental health risks. Then we identified a range of potential interventions which could alleviate these risks including sexual and reproductive health interventions, nutrition interventions, infections and immunizations, mental health interventions, substance abuse, and injury prevention interventions. The conceptual framework shows that implementation of these interventions could yield immediate and direct results, including improving access to sexual health, mental health, and substance abuse services; knowledge of sexually transmitted infections, dietary behavior, and physical activity; immunization uptake; and delivery of suicide preventive services. Broadly, the conceptual framework classifies outcomes to individual, community, and societal levels, and it illustrates that the immediate and direct impacts could yield improved health, better adult life, and improved work productivity; these individual impacts could lead to gains at the family and immediate community which collectively could help accelerate economic growth and national progress.

In this article, we conducted a comprehensive overview of systematic reviews for the effectiveness of substance abuse interventions for adolescents and various delivery platforms.

## Methods

We systematically reviewed literature published up to December 2015 to identify systematic reviews on interventions for substance abuse in adolescent population. For the purpose of this overview, the adolescent population was defined as aged 11–19 years; however, since many reviews targeted youth (aged 15–24 years) along with adolescents, exceptions were made to include reviews targeting adolescents and youth. We did not apply any limitations on the start search date or geographical settings. We considered all available published systematic reviews on interventions for adolescent substance abuse. A broad search strategy was used that included a combination of appropriate keywords, medical subject heading, and free text terms. Search was conducted in the Cochrane Library and PubMed. The abstracts (and the full sources where abstracts are not available) were screened by two abstractors to identify systematic reviews adhering to our objectives. Any disagreements on selection of reviews between these two primary abstractors were resolved by the third reviewer. After retrieval of the full texts of all the reviews that met the inclusion/exclusion criteria, data from each review were extracted independently into a standardized form. Information was extracted on (1) the characteristics of included studies; (2) description of methods, participants, interventions, and outcomes; (3) measurement of treatment effects; (4) methodological issues; and (5) risk of bias tool. We extracted pooled effect size for the outcomes reported by the review authors with 95% confidence intervals (CIs). We assessed and reported the quality of included reviews using the 11-point assessment of the methodological quality of systematic reviews (AMSTAR) criteria [31]. We excluded nonsystematic reviews, nonindexed publications/reports, systematic reviews evaluating the efficacy of pharmacological intervention, systematic reviews focusing on interventions for secondhand smoking, systematic review focusing on multiple health risk factors rather than substance abuse alone, systematic reviews focusing on specific population groups (e.g., European countries) alone, interventions targeting population other than adolescents and youth, and reviews not reporting outcomes related to substance abuse.

## Results

**Figure 1:**
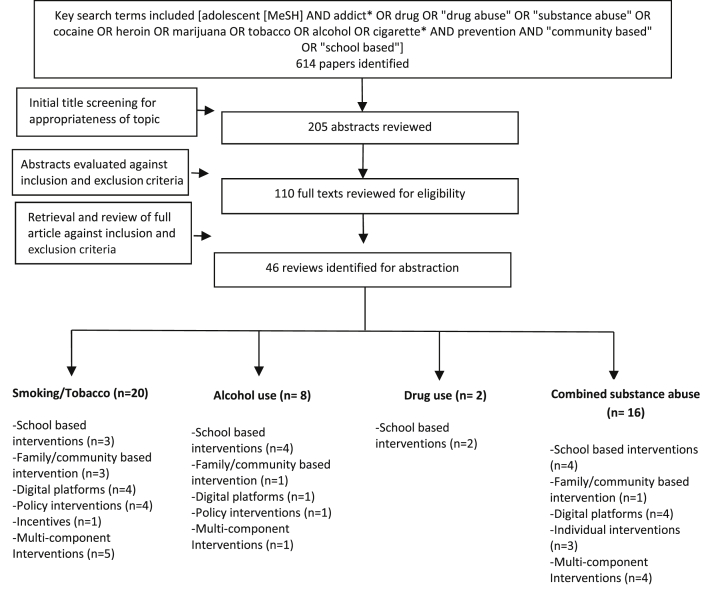
Search flow diagram. MeSH = Medical Subject Heading.

Our search identified 614 potentially relevant review titles, of which 110 full texts were reviewed. Finally, 46 reviews were deemed eligible and meeting the inclusion criteria (Figure 1). We classified the included reviews into the following categories for reporting findings:

1. Intervention for smoking/tobacco use (n = 20)
2. Interventions for alcohol use (n = 8)
3. Interventions for drug use (n = 2)
4. Interventions targeting combined substance abuse (n = 16)

Table 1 describes the characteristics of the included reviews while Table 2 provides the summary estimates for all the interventions.

### Interventions for smoking/tobacco use

We report findings from a total of 20 systematic reviews focusing on various interventions for smoking/tobacco use among adolescents. Of these 20 reviews, three reviews focused on school-based interventions; three reviews focused on family-/community-based interventions; four reviews focused on digital platforms; four reviews focused on policy interventions; one review focused on the effect of providing incentives; while five reviews focused on multicomponent interventions for smoking/tobacco use among adolescent age group. The AMSTAR rating for the reviews ranged between 5 and 10 with a median score of 8. Meta-analysis was conducted in nine of the included reviews.

#### School-based interventions

We report findings from three systematic reviews focusing on school-based interventions for smoking/tobacco use among adolescents [32], [33], [34]. A review based on 134 studies evaluated the impact of school smoking interventions for preventing youth from starting smoking [32] and suggested that pure prevention program (where never-smokers at baseline were followed and the number of remaining never-smokers at the various follow-up intervals was ascertained), and combined social competence and social influences curricula have an overall significant effect on reducing smoking initiation (relative risk [RR]: .88; 95% CI: .82–.96 and RR: .49; 95% CI: .28–.87, respectively) while there is no impact of only-information or social influence interventions. Another review evaluated the impact of “Smoke-Free Class Competition” (SFC) [33]. SFC is a school-based smoking prevention program including commitment not to smoke, contract management, and prizes as rewards. Findings from this review suggest that SFC participation is effective in reducing students who are currently smoking (RR: .86; 95% CI: .79–.94). A review specifically focused on long-term follow-up of school-based smoking prevention trials and reported that the interventions varied in intensity, presence of booster sessions, follow-up periods, and attrition rates. This review found very limited evidence on long-term impact of school-based smoking prevention programs [34].

#### Family-/community-based interventions

We included three systematic reviews evaluating the impact of family-/community-based interventions for smoking/tobacco use among adolescents [35], [36], [37]. Family-based interventions had a positive impact on preventing smoking with a significant reduction in smoking behavior (RR: .76; 95% CI: .68–.84) [35]. Most of these studies used intensive interventions typically addressing family functioning and introduced when children were between 11 and 14 years old. However, these findings should be interpreted cautiously because effect estimates could not include data from all studies. Another review evaluated the impact of coordinated widespread community interventions which support nonsmoking behavior [36]. The interventions included involvement of community leaders for the development and support of community programs, training community workers to form a community coalition of diverse stakeholders to implement and monitor smoking prevention interventions, and involving multiple organizations including the national health service, city councils, social workers, business owners, voluntary organizations, sports organizations, health care providers, community organizations, media, retailers, schools, government, law enforcement, or workplaces. Findings from 25 studies suggest positive impact of community-delivered interventions on reducing smoking rates, intentions to smoke, and increasing knowledge about effects of smoking; however, the evidence is not strong and contains a number of methodological flaws [36]. Evidence from primary care relevant interventions (including coordinated, multicomponent interventions that combine mass media campaigns, price increases, school-based policies and programs, and statewide or community-wide changes in policies and norms) suggests a significant reduction in smoking initiation (RR: .81; 95% CI: .70–.93) among participants in behavior-based prevention interventions with no impact on cessation rates [37]. However, the interventions and measures were reported to be heterogeneous.

#### Digital platforms

We report findings from four systematic reviews evaluating various digital platforms for smoking/tobacco use among adolescent age group [38], [39], [40], [41]. A review evaluating antitobacco mass media campaigns suggests that these media campaigns can be effective across various racial/ethnic populations for smoking prevention, although the size of the campaign effect may differ by race/ethnicity [39]. Existing evidence supports advertising that includes personal testimonials; surprising narrative; and intense images, sound, and editing while research is insufficient to determine whether advertising with secondhand smoke or social norms theme influences youth tobacco use. Another review evaluated the effectiveness of mass media interventions to prevent smoking in young people in terms of reduced smoking uptake, improved smoking outcomes, attitudes, behaviors, knowledge, self-efficacy, and perception [41] and suggests that mass media can prevent the uptake of smoking in young people; however, the evidence is not strong and contains a number of methodological flaws. The review further suggests that effective media campaigns had a solid theoretical basis, used formative research in designing the campaign messages, and message broadcast was of reasonable intensity over extensive period of time. A review on Web-based smoking cessation interventions among college students suggests mixed results, with insufficient evidence supporting their efficacy [38]. Another review evaluating Internet-based interventions for smoking cessation suggests that Internet-based interventions can assist smoking cessation for a period of 6 months or longer, particularly those which were interactive and tailored to individuals; however, more research is needed to confirm the findings [40].

#### Policy level interventions

We found four reviews reporting the impact of smoking/tobacco use policy initiatives [42], [43], [44], [45]. A review evaluating the effect of tobacco advertising and promotion suggests that these policies increase the likelihood of adolescents to start smoking [42]. However, there was variation in the strength of association and the degree to which potential confounders were controlled for. A review evaluated the impact of school policies aiming to prevent smoking initiation [43] and included only one trial. The review suggests no difference in smoking prevalence between intervention and control schools. One review assessed the effect of interventions to reduce underage access to tobacco by deterring shopkeepers from making illegal sales [44]. This review suggests that giving retailer's information is less effective in reducing illegal sales than active enforcement and/or multicomponent educational strategies while there is little effect of intervention on youth perceptions of access to tobacco products or prevalence of youth smoking. Another review evaluated the effectiveness of laws restricting youth access to cigarettes by limiting the ability of teens to purchase cigarette on prevalence of smoking among teens [45]. Findings suggest that there is no detectable relationship between the level of merchant compliance and 30-day or regular smoking prevalence and no significant difference in youth smoking.

#### Incentives

We found one review evaluating the impact of incentives (involving any tangible benefit externally provided with the explicit intention of preventing smoking. This includes contests, competitions, incentive schemes, lotteries, raffles, and contingent payments to reward not starting to smoke) to prevent smoking among adolescents [46]. Findings from seven included trials suggest that there is no statistically significant effect of incentives to prevent smoking initiation among children and adolescents (RR: 1.00; 95% CI: .84–1.19). There is lack of robust evidence to suggest that unintended consequences (such as youth making false claims about their smoking status and bullying of smoking students) are consistently associated with such interventions, although this has not been the focus of much research. There was insufficient information to assess the dose–response relationship or costs.

#### Multicomponent interventions

We found five reviews addressing multicomponent interventions for smoking/tobacco use among adolescents [47], [48], [49], [50], [51]. One review evaluated the long-term effectiveness of different school-based, community-based, and multisectorial intervention strategies [47]. Although the overall effectiveness of prevention programs showed considerable heterogeneity, the majority of studies report some positive long-term effects for behavioral smoking prevention programs. There was evidence that community-based and multisectoral interventions were effective in reducing smoking rates, while the evidence for school-based programs alone was inconclusive. Another review evaluating any intervention for smoking cessation suggests that any type of intervention is more effective in producing successful smoking cessation compared to no intervention (RR: 1.55; 95% CI: 1.16–2.06) [48]. One review evaluated the effectiveness of strategies that help young people to stop smoking tobacco [49]. Majority of the included studies used some form of motivational enhancement combined with psychological support such as cognitive behavioral therapy (CBT), and some were tailored to stage of change using the transtheoretical model. Transtheoretical model and motivational enhancement interventions have shown moderate long-term success (RR: 1.56; 95% CI: 1.21–2.01) and (RR: 1.60; 95% CI: 1.28–2.01), respectively. However, complex interventions that included CBT did not achieve statistically significant results. A review evaluating interventions targeting smoking cessation among adolescents suggests limited evidence demonstrating efficacy of smoking cessation interventions in adolescents and no evidence on the long-term effectiveness of such interventions [50]. One review specifically evaluated the effectiveness of intervention programs to prevent tobacco use, initiation, or progression to regular smoking amongst young indigenous populations [51]. The review included two studies reporting no difference in weekly smoking at 42-month follow-up.

### Interventions for alcohol use

We report findings from a total of eight systematic reviews focusing on various interventions for alcohol use among adolescents. Four reviews focused on school-/college-based interventions while one review each focused on family-/community-based interventions, digital platforms, policy interventions, and multicomponent interventions. The AMSTAR rating ranged between 7 and 10 with a median score of 8.5. Meta-analysis was conducted in five of the included reviews.

#### School-based interventions

We report findings from a total of four reviews focusing on school-/college-based interventions for alcohol use [52], [53], [54], [55]. A review evaluating college-based interventions for alcohol misuse prevention suggests lower quantity and frequency of drinking and fewer problems among the adolescents in the intervention group compared to controls [52]. Findings suggest that college-based interventions that include personalized feedback, moderation strategies, expectancy challenge, identification of risky situations, and goal setting are effective in reducing alcohol-related behavior issues among adolescents. Another review evaluating school-based prevention program showed that, overall, the effects of school-based preventive alcohol interventions on adolescent alcohol use were small but positive among studies reporting the continuous measures, whereas no effect was found among studies reporting the categorical outcomes [53]. School-based brief alcohol interventions (BAIs) among adolescents are associated with significant reduction in alcohol consumption [54]. Subgroup analyses indicated that individually delivered BAIs are effective while there is no evidence that group-delivered BAIs are also associated with reductions in alcohol use. Universal school-based preventive interventions showed some evidence of effectiveness compared to a standard curriculum [55].

#### Family-/community-based interventions

We found one review evaluating the impact of universal family-based prevention programs (including any form of supporting the development of parenting skills including parental support, nurturing behaviors, establishing clear boundaries or rules, and parental monitoring) in preventing alcohol misuse in school-aged adolescents [56]. Most of the trials in the included review have shown some evidence of effectiveness, with persistence of effects over the medium and longer term. The review concluded that the effects of family-based prevention interventions are small but generally consistent and also persistent over the medium to long term.

#### Digital platforms

We found one systematic review reporting the efficacy of computer-delivered interventions (CDIs) to reduce alcohol use among college students [57]. The typical intervention was a single-session computerized task delivered via the Internet, intranet, or CD-ROM/DVD lasting a median of 20 minutes. Most CDIs were delivered on-site, whereas some of students completed the CDI off-site. The effects of CDIs depended on the nature of the comparison condition: CDIs reduced quantity and frequency measures relative to assessment-only controls but rarely differed from comparison conditions that included alcohol-relevant content. Overall, CDIs are found to reduce the quantity and frequency of drinking among college students and are comparable to alternative alcohol-related comparison interventions.

#### Policy interventions

We found one review that evaluated restriction or banning of alcohol advertising via any format including advertising in the press, on the television, radio, Internet, billboards, social media, or product placement in films [58]. The review found lack of robust evidence for or against recommending the implementation of alcohol advertising restrictions. Advertising restrictions should be implemented within a high-quality, well-monitored research program to ensure the evaluation over time of all relevant outcomes in order to build the evidence base.

#### Multicomponent interventions

We found one review evaluating the effectiveness of universal multicomponent prevention programs in preventing alcohol misuse in school-aged children [59]. Twelve of the 20 trials showed evidence of effectiveness, with persistence of effects ranging from 3 months to 3 years. There is some evidence that multicomponent interventions for alcohol misuse prevention in young people can be effective. However, there is little evidence that interventions with multiple components are more effective than interventions with single component.

### Interventions for drug use

We report findings from two systematic reviews focusing on various interventions for drug use among adolescents. Both the reviews focused on school-based interventions. The AMSTAR rating for the reviews ranged between 8 and 10 with a median score of 9. Meta-analysis was conducted in both the included reviews.

#### School-based interventions

We found two reviews evaluating school-based interventions for drug use [60], [61]. One review evaluated school-based primary prevention interventions including educational approaches (knowledge-focused, social competence–focused, and social norms–focused programs; combined programs; other types of interventions). Findings suggest that both social influence and social competent approach combined favors intervention (RR: .83; 95% CI: .69–.99) for marijuana use at 12+ months with no difference on hard drug use at 12+ months (RR: .86; 95% CI: .39–1.90). Combined interventions are effective in reducing any drug use at <12 months (RR: .76; 95% CI: .64–.89). Overall, school programs based on a combination of social competence and social influence approaches have shown, on average, small but consistent protective effects in preventing drug use. Another review evaluating the impact of school-based programs on cannabis use suggested that school-based programs have a positive impact on reducing students' cannabis use compared to control conditions [61]. Findings revealed that programs incorporating elements of several prevention models were significantly more effective than those were based only on a social influence model. Programs that were longer in duration (≥15 sessions) and facilitated by individuals other than teachers in an interactive manner also yielded stronger effects.

### Interventions for combined substance abuse

We report findings from a total of 16 systematic reviews focusing on various interventions for combined substance abuse among adolescents. Of these 16 reviews, four reviews focused on school-based interventions, one review focused on family-/community-based interventions, four reviews focused on digital platforms, three reviews focused on individual-targeted interventions (mentoring and psychotherapy), and four reviews focused on multicomponent interventions. The AMSTAR rating for the reviews ranged between 6 and 10 with a median score of 7. Meta-analysis was conducted in five of the included reviews.

#### School-based interventions

We found four systematic reviews evaluating the impact of school-based interventions targeting substance abuse among adolescents [62], [63], [64], [65]. Interventions that promote a positive school ethos and reduce student disaffection may be an effective complement to drug prevention interventions addressing individual knowledge, skills, and peer norms [65]. One review based on 18 program evaluations suggested mixed and inconclusive evidence to provide any judgment on the effectiveness of school-based programs [62]. Another review evaluating the effectiveness of brief school-based interventions in reducing substance use and other behavioral outcomes among adolescents found moderate quality evidence that, compared to information provision only, brief interventions did not have a significant effect on any of the substance use outcomes at short-, medium-, or long-term follow-up [63]. When compared to assessment-only controls, brief interventions reduced cannabis frequency, alcohol use, alcohol abuse and dependence, and cannabis abuse. Brief interventions also have mixed effects on adolescents' delinquent or problem behaviors, although the effect at long-term follow-up on these outcomes in the assessment-only comparison was significant. School-based marijuana and alcohol prevention programs are found to be effective in preventing marijuana and alcohol use in adolescents between the ages of 10 and 15 years [64]. The most effective primary prevention programs for reducing marijuana and alcohol use among adolescents aged 10–15 years in the long term are comprehensive programs that included antidrug information combined with refusal skills, self-management skills, and social skills training.

#### Family-/community-based interventions

We found one review evaluating parenting programs to prevent tobacco, alcohol, or drug abuse in children younger than 18 years [66]. Findings suggest that parenting programs can be effective in reducing or preventing substance use. The most effective intervention appears to be those that shared an emphasis on active parental involvement and on developing skills in social competence, self-regulation, and parenting. However, more work is needed to investigate further the change processes involved in such interventions and their long-term effectiveness.

#### Digital platforms

We report findings from four reviews evaluating digital platforms for substance abuse among adolescents [67], [68], [69], [70]. A review evaluating the impact of Internet-based programs and intervention delivered via CD-ROM targeting alcohol, cannabis, and tobacco suggests that these programs have the potential to reduce alcohol and other drug use as well as intentions to use substances in the future [67]. Web-based interventions for problematic substance use by adolescents and young adults highlighted insufficient data to assess the effectiveness of Web-based interventions for tobacco use by adolescents [68]. For Internet and mobile phone use, one review suggested good empirical evidence concerning the efficacy of Web-based social norms interventions to decrease alcohol consumption in students [69]. Internet interventions for smoking prevention are found to be heterogeneous. Interventions using mobile phone text messaging for smoking cessation are found to be well accepted and promising; however, they are primarily tested within pilot studies, and conclusions about their efficacy are not possible so far. One review evaluated the impact of serious educational games targeting tobacco, alcohol, cannabis, methamphetamine, ecstasy, inhalants, cocaine, and opioids and reported very limited evidence to suggest benefit [70].

#### Individual-targeted interventions

We report findings from three systematic reviews evaluating individual-targeted interventions for substance abuse among adolescents; these included mentoring [71], counseling, or psychotherapy [72], [73]. Review evaluating mentoring suggested limited evidence to conclude that the intervention was effective [71]. The review evaluating counseling and psychotherapy to treat alcohol and other drug use problems in school-aged youth suggested that the effects of counseling and psychotherapy for drug abuse are consistently significant at termination, but follow-up effects yielded inconsistent results [72]. A review evaluating CBT, family therapy replication, and minimal treatment control conditions suggested the need for more data since none of the treatment approaches appeared to be clearly superior to any others in terms of treatment effectiveness for adolescent substance abuse [73].

#### Multicomponent intervention

We report findings from four systematic reviews evaluating multicomponent interventions for substance abuse among adolescents [74], [75], [76], [77]. One review suggested that there is some empirical evidence of the effectiveness of social influences programs in preventing or reducing substance use for up to 15 years after completion of programming. However, this conclusion is prone to great variability in the level of internal and external validity across all studies [74]. Another review suggested that multidimensional family therapy and cognitive behavioral group treatment received the highest level of evidentiary support [75]. Early interventions for adolescent substance use do hold benefits for reducing substance use and associated behavioral outcomes if delivered in an individual format and over multiple sessions [76]. One review found relatively few studies on the adolescent substance abuse treatment and suggested that there is evidence that treatment is superior to no treatment but insufficient evidence to compare the effectiveness of treatment types [77].

## Discussion

We included 46 systematic reviews focusing on interventions for smoking/tobacco use, alcohol use, drug use, and combined substance abuse. Our overview findings suggest that among smoking/tobacco use interventions, school-based pure prevention programs and SFC are effective in reducing smoking initiation and current smoking. However, there is lack of long-term follow-up for the impact of school-based smoking/tobacco use programs. Family-based intensive interventions typically addressing family functioning are also found to effectively prevent smoking. Coordinated widespread community-based interventions have also shown positive impacts on smoking behaviors. Mass media campaigns involving solid theoretical basis, formative research in designing the campaign messages, and message broadcast have shown positive impacts on uptake of smoking given that these were of reasonable intensity over extensive periods of time. Evidence from Internet-based interventions, policy initiatives, and incentives appears to be mixed and needs further research.

Among interventions for alcohol use, school-based alcohol prevention interventions including personalized feedback, moderation strategies, expectancy challenge, identification of risky situations, goal setting, and BAIs have been associated with reduced frequency of drinking. Family-based interventions have a small but persistent effect on alcohol misuse among adolescents while CDIs for alcohol are found to reduce the quantity and frequency of drinking among college students. There is lack of robust evidence for or against recommending the implementation of alcohol advertising restrictions and multiple component interventions. For drug use, school-based interventions based on a combination of social competence and social influence approaches have shown protective effects in preventing drugs and cannabis use. Among the interventions targeting combined substance abuse, school-based primary prevention programs that include antidrug information combined with refusal skills, self-management skills, and social skills training are effective in reducing marijuana and alcohol use among adolescents. There is very limited evidence on the effectiveness of mass media and mentoring for combined substance abuse.

We adopted an overview of reviews approach for synthesizing existing evidence on adolescent substance abuse. Although an overview of systematic reviews builds on the conclusions of rigorous reviews of studies in different settings and of varying quality, avoids duplication of work and allows for a much faster review, there are some potential limitations. The interventions on which primary data exist, but which have not been covered by a systematic review, will not have been included. Furthermore, an overview of systematic reviews relies on review authors' characterizations of the findings rather than on individual studies and therefore may be affected by selective reporting biases. It also misses upon studies not taken up by included reviews. However, we have quality rated the existing reviews for transparency.

Our review findings highlight that school-based delivery platforms are the most highly evaluated platforms for targeting adolescents for substance abuse. Most of the existing evidence for substance abuse interventions comes from HICs. There is lack of data to determine the differential effects of interventions by gender, socioeconomic status, and population density. Meta-analysis could not be conducted in most of the included reviews since the interventions varied in intensity, follow-up periods, and reported outcomes. Furthermore, in reviews where meta-analysis was conducted, not all the data contributed to the pooled effect estimate. There is lack of rigorous data evaluating the sustainability and long-term effectiveness of substance abuse programs targeting adolescents. Future research should focus on evaluating the effectiveness of specific intervention components with standardized intervention and outcome measures. There is a need to evaluate relative effectiveness and cost-effectiveness of various delivery platforms targeting adolescents for substance abuse interventions. Various delivery platforms, including digital platforms and policy initiative, have the potential to improve substance abuse outcomes among adolescents; however, these require further research. Future trials should focus on reporting separate data for gender and socioeconomic subgroups since the impact of such behavior change interventions might vary among various population subgroups. Lastly, there is a dire need for rigorous, higher quality evidence especially from low- and middle-income countries on effective interventions to prevent and manage substance abuse among adolescents.

## Figures and Tables

**Table 1:** Characteristics of included reviews

Intervention	Review	Number of included studies	Setting	Intervention details	AMSTAR rating	Meta-analysis	Outcomes reported
Smoking/tobacco							
School-based interventions	Thomas et al. [32]	134 RCTs	Mostly in high-income countries except a few trials in India, Thailand, and Mexico	Information-only curricula, social competence curricula, social influence curricula, multimodal programs	9	Yes	Smoking status
	Isensee and Hanewinkel [33]	5 RCTs	High-income countries	“Smoke-Free Class competition” (SFC) is a school-based smoking prevention program including commitment not to smoke, contract management, and prizes as rewards broadly implemented in Europe.	6	Yes	Current smoking at follow-up
	Wiehe et al. [34]	8 RCTs	High-income countries	School-based smoking prevention trials with follow-up smoking prevalence data through at least 12th grade or age 18 years	6	No	Smoking prevalence
Family-/community-based interventions	Thomas et al. [35]	27 RCTs	All in high-income countries except one in India	Interventions with children and family members intended to deter starting to use tobacco. Those with school- or community-based components were included provided the effect of the family-based intervention could clearly be measured and separated from the wider school- or community-based interventions. Interventions that focused on preventing drug or alcohol use were included if outcomes for tobacco use were reported. The family-based intervention could include any components to change parenting behavior, parental or sibling smoking behavior, or family communication and interaction.	10	Yes	New smoking at follow-up, smoking at follow-up
	Carson et al. [36]	15 RCTs and 10 CCTs	All in high-income countries except one in India	Interventions were considered which (1) were targeted at entire or parts of entire communities or large areas, (2) had the intention of influencing the smoking behavior of young people, and (3) focused on multicomponent (i.e., more than one) community intervention, which could include but was not limited to: school-based programs, media promotion (e.g., TV, radio, print), public policy, organizational initiatives, health care provider initiatives, sports, retailer and workplace initiatives, antitobacco contests, and youth antismoking clubs. Community interventions were defined as coordinated widespread (multicomponent) programs in a particular geographical area (e.g., school districts) or region or in groupings of people who share common interests or needs, which support nonsmoking behavior. Studies which only included single component interventions, did not have community involvement (e.g., school based only), or had mass media as the sole form of intervention delivery were excluded.	10	Yes	Smoking daily, smoking weekly, smoking monthly, ever smoked, smokeless tobacco use
	Patnode et al. [37]	19 RCTs	All in high-income countries	Primary care interventions	5	Yes	Smoking initiation, smoking cessation
Digital platforms	Hutton et al. [38]	21 RCTs	All in high-income countries	Web delivered smoking cessation program and had a minimum of 1-month follow-up after intervention.	8	No	Smoking cessation
	Allen et al. [39]	—	—	Antitobacco media campaign intended to influence youth cognitions or behavior or explore the relative effectiveness of campaign characteristics among youth.	—	No	—
	Civljak et al. [40]	28 RCTs and quasi RCTs	All in high-income countries	Internet-based interactive, personalized and noninteractive interventions, which focused on standard approaches to information delivery. Interactive interventions were not necessarily personalized.	9	No	Smoking cessation at 6 months
	Brinn et al. [41]	7 RCTs	All in high-income countries	Mass media is defined here as channels of communication such as television, radio, newspapers, billboards, posters, leaflets, or booklets intended to reach large numbers of people and which are not dependent on person-to-person contact.	9	No	Smoking/tobacco use status
Policy interventions	Lovato et al. [42]	19 longitudinal studies	All in high-income countries	The “intervention” is tobacco mass media advertising by the industry, including tobacco promotion. Mass media channels of communication include advertising delivered through television, radio, newspapers, billboards, posters, and so forth. Tobacco promotion includes giveaways such as T-shirts and other items bearing tobacco industry logos. In practice, the measure of exposure to the intervention may not discriminate between specific types of advertising since adolescents are exposed to many sources. Indices of receptivity to advertising which use measures such as having a favorite advertisement, and ownership of or willingness to own promotional items could be used as indicators of exposure.	6	No	Self-reported smoking status (nonsmoker, current smoker, ex-smoker) Self-reported consumption of specific brands
	Coppo et al. [43]	1 RCT	China	All written policies that regulate tobacco use inside and/or outside the school property were eligible. We would have classified interventions as partial bans, inside bans, and comprehensive policies. We would have included studies of policies aiming to ban drug or alcohol use in addition to smoking if tobacco use outcomes were reported. We would have considered interventions in which an STP was a component of a smoking prevention program only if it was possible to isolate its effect. Studies that compared stronger and weaker policies were eligible. We would have considered whether the implementation of a policy had an impact on its effect.	10	Not applicable	Prevalence of current smokers
	Stead and Lancaster [44]	35 studies	All in high-income countries	The main interventions were education about legal requirements, notification of the results of compliance checks, warning of enforcement, and implementation of enforcement by police or health officials.	8	No	1. Illegal tobacco sales, assessed by attempted purchase by young people. 2. Perceived ease of access to cigarettes by young people. 3. Prevalence of tobacco use among young people. We accepted self-reports of tobacco use.
	Fichtenberg and Glantz [45]	9 studies	All in high-income countries	Presence of restrictions on the ability of teens to purchase cigarettes	7	Yes	30-day smoking prevalence, regular smoking prevalence
Incentives	Thomas and Johnston [46]	7 cRCTs	High-income countries	An incentive was any tangible benefit externally provided with the explicit intention of preventing smoking. This includes contests, competitions, incentive schemes, lotteries, raffles, and contingent payments to reward not starting to smoke. We included rewards to third parties (e.g., to schools, health care providers, or family members), as well as interventions that directly reward children and adolescents.	9	Yes	Smoking uptake at longest follow-up
Multicomponent interventions	Müller-Riemenschneider et al. [47]	35 RCTs	All in high-income countries except one in India	A mixture of school-based, community-based and multicomponent interventions	8	Yes	Lifetime smoking, 30-day smoking, regular smoking
	Suls et al. [48]	14 studies	All in high-income countries	Any smoking cessation interventions	6	Yes	Smoking cessation
	Stanton and Grimshaw [49]	28 RCTs	All in high-income countries	Interventions could be specifically designed to meet the needs of young people aged <20 years or could also be applicable to adults. Interventions could range from simple ones such as pharmacotherapy, targeting individual young people, through strategic programs targeting people, or organizations associated with young people (for example, their families or schools), to complex programs targeting the community in which young people study or live.	9	Yes	Smoking cessation
	Garrison et al. [50]	6 RCTs	All in high-income countries except one in Singapore	Any intervention targeting adolescent smoking cessation	7	No	Smoking cessation
	Carson et al. [51]	2 RCTs	All in high-income countries	Interventions considered in this review aim to prevent tobacco use initiation or progression from experimentation to regular tobacco use in indigenous youth.	9	No	Tobacco use
Alcohol use							
School-based interventions	Scott-Sheldon et al. [52]	41 studies	All in high-income countries	Interventions were typically delivered during a single-session lasting less than 1 hour. Most interventions were delivered to individuals, but some were delivered in groups and others used a combination of individual and group sessions.	8	Yes	Alcohol consumption and alcohol-related problems
	Strøm et al. [53]	28 RCTs	All in high-income countries	Any school-based programs targeting alcohol misuse	8	Yes	Alcohol use
	Hennessy and Tanner-Smith [54]	17 RCTs and quasi	All in high-income countries	School-based individual or group-delivered interventions using a range of modalities (motivational enhancement therapy; cognitive behavioral therapy/skills training; cognitive behavioral and motivational enhancement therapy combined; psychoeducational therapy) whereas all the individually delivered interventions used an MET approach.	7	Yes	Alcohol use
	Foxcroft and Tsertsvadze [55]	53 RCTs	Mostly in high-income countries except one in India and one in Swaziland	Universal school-based psychosocial or educational prevention program; psychosocial intervention is defined as one that specifically aims to develop psychological and social skills in young people (e.g., peer resistance) so that they are less likely to misuse alcohol; educational intervention is defined as one that specifically aims to raise awareness of the potential dangers of alcohol misuse so that young people are less likely to misuse alcohol; studies that evaluated interventions aiming specifically at preventing and reducing alcohol misuse as well as generic interventions (e.g., drug education programs, healthy school or community initiatives) or other types of interventions (e.g., screening for alcohol consumption) were eligible for inclusion in the review.	9	No	Alcohol use
Family-/community-based interventions	Foxcroft and Tsertsvadze [56]	12 RCTs		Any universal family-based psychosocial or educational prevention program. Psychosocial intervention is defined as one that specifically aims to develop psychological and social attributes and skills in young people (e.g., behavioral norms, peer resistance), via parental socialization and influence, so that young people are less likely to misuse alcohol. Educational intervention is defined as one that specifically aims to raise awareness amongst parents and/or carers of how to positively influence young people or of the potential dangers of alcohol misuse, so that young people are less likely to misuse alcohol. Studies that evaluated interventions aiming specifically at preventing and reducing alcohol misuse as well as generic interventions (e.g., drug education programs) or other types of interventions (e.g., screening for alcohol consumption) were eligible for inclusion in the review.	9	No	Alcohol consumption
Digital platforms	Carey et al. [57]	35 studies	All in high-income countries	The typical intervention was a single-session computerized task delivered via the Internet, intranet, or CD-ROM/DVD lasting a median of 20 minutes. Most CDIs were delivered on-site, whereas some of the students completed the CDI off-site.	8	Yes	Alcohol consumption and problems
Policy interventions	Siegfried et al. [58]	2 studies (1 RCT and 3 ITSs)	All in high-income countries	Studies that evaluated the restriction or banning of alcohol advertising via any format including advertising in the press, on the television, radio, or Internet, via billboards, social media, or product placement in films.	10	Yes	Alcohol consumption, alcohol sales
Multicomponent interventions	Foxcroft and Tsertsvadze [59]	20 RCTs	All in high-income countries except one in India	Universal multicomponent prevention programs in preventing alcohol misuse in school-aged children up to 18 years. Multicomponent prevention programs are defined as those prevention efforts that deliver interventions in multiple settings, for example, in both school and family settings, typically combining school curricula with a parenting intervention.	10	No	Alcohol use
Drug use							
School-based interventions	Faggiano et al. [60]	51 RCTs	All in high-income countries	School-based primary prevention interventions, classified in terms of their: • educational approaches (knowledge-focused, social competence–focused and social norms–focused programs, combined programs, other types of interventions); • targeted substances (we included programs addressing all substances including alcohol but only extracted outcomes related to illicit substance use); • type of setting (we excluded interventions combining school-based programs with extra school programs).	10	Yes	Marijuana use, hard drug use, any drug use
	Porath-Waller et al. [61]	15 RCTs	All in high-income countries	School-based programs targeting cannabis use among adolescents	8	Yes	Cannabis use
Interventions targeting combined substance abuse							
School-based interventions	Manoj Sharma et al. [62]	18 studies	All in high-income countries except one in China	School-based interventions for preventing any substance abuse	6	No	Drug use
	Carney et al. [63]	6 RCTs	All in high-income countries	Brief interventions (BIs) are targeted, time-limited, low-threshold services that aim to reduce substance use and its associated risks, as well as prevent progression to more severe levels of use and potential negative consequences.	10	Yes	Alcohol frequency, alcohol quantity, cannabis dependence, cannabis frequency, other substance abuse related outcomes
	Lemstra et al. [64]	6 RCTs	All in high-income countries	School-based interventions to prevent marijuana and/or alcohol use (defined as at least once per month) in adolescents between the ages of 10 and 15 years old.	8	Yes	Knowledge, alcohol use, marijuana use
	Fletcher et al. [65]	4 trials	All in high-income countries	School institutional factors influence young people's use of drugs	6	No	
Family-/community-based interventions	Petrie et al. [66]	20 RCTs	All in high-income countries	“Parenting programs” as any intervention involving parents which was designed to develop parenting skills, improve parent/child communication, or enhance the effects of other interventions, for example, classroom-based programs. We included all types of learning medium, for example, group discussion, distance learning by the Internet or post, video program, individual coaching, and so forth, and any source of delivery, for example, programs provided by health visitors or school nurses, programs run by charities or voluntary organizations, and so forth. Interventions where there was minimal contact with parents (e.g., leaflets only) were not considered to constitute a program and were therefore excluded.	8	No	Any substance abuse or intent for substance abuse
Digital platforms	Champion et al. [67]	12 RCTs	All in high-income countries	Seven trials evaluated Internet-based programs and five delivered an intervention via CD-ROM. The interventions targeted alcohol, cannabis, and tobacco.	8	No	Alcohol, cannabis, and tobacco use
	Tait and Christensen [68]	16 RCTs	All in high-income countries	Web-based interventions	7	No	Substance abuse
	Haug et al. [69]	31 studies	All in high-income countries	Internet and mobile phone interventions to decrease alcohol consumption and for smoking cessation in adolescents	7	No	Substance abuse
	Rodriguez et al. [70]	8 studies	All in high-income countries	Serious educational games	7	No	Knowledge
Individual interventions	Thomas et al. [71]	4 RCTs	All high-income countries	All mentoring programs whose goal is to deter alcohol and drug use, irrespective of theoretical intervention	9	Yes	Alcohol use, substance use, marijuana use
	Rongione et al. [72]	20 studies	All high-income countries	The definition of counseling or psychotherapy for substance abuse was any intervention or treatment used to reduce substance use and provided by a mental health professional or professional-in-training.	7	No	Substance abuse frequency
	Waldron and Turner [73]	17 studies	All high-income countries	Cognitive behavioral therapy (CBT), family therapy replications, minimal treatment control conditions	7	No	Substance abuse frequency
Multicomponent interventions	Skara and Sussman [74]	25 studies	All high-income countries	Prevention strategies that addressed the issues of social influences to smoke and the development of skills to resist such pressures	7	No	Frequency of substance use
	Vaughn and Howard [75]	18 studies	All high-income countries	Multidimensional interventions: family-based, psychotherapy, education, behavioral therapy, life skills training	7	No	Substance abuse
	Carney and Myers [76]	9 RCTs	All high-income countries	Early interventions that target adolescent substance use as a primary outcome, and criminal or delinquent behaviors as a secondary outcome	8	Yes	Aggregate effect estimate
	Williams and Chang [77]	53 studies	Mostly high-income countries	Comprehensive range of treatment (individual counseling, group therapy, medication for comorbid conditions, family therapy, schooling, and recreational programming)	7	Yes	Alcohol frequency, binge drinking, marijuana use

AMSTAR = assessment of the methodological quality of systematic reviews; CCT = controlled clinical trial; CDI = computer-delivered intervention; cRCT = cluster randomized controlled trial; ITS = interrupted tie series; MD = mean difference; MET = motivational enhancement therapy; RCT = randomized controlled trials; RD = risk difference; STP = school tobacco policies.

**Table 2:** Summary estimates for substance abuse interventions

Substance abuse	Interventions	Outcomes and estimates
Smoking/tobacco use	School-based interventions	**Smoking uptake (pure prevention; RR: .88; 95% CI: .82–.96)** **Smoking at follow-up (smoke-free class competition; RR: .86; 95% CI: .79–.94)** **Smoking prevalence (at long-term follow-up) (RD: −.61; 95% CI: −4.22 to 3.00)**
	Family-/community-based interventions	**New smoking at follow-up (baseline never-smokers; RR: .76; 95% CI: .68–.84)** *Smoking at follow-up (baseline smoking not restricted; RR: 1.04; 95% CI: .93–1.17)* *Weekly smoking (RR: .83; 95% CI: .59–1.17)* *Monthly smoking (RR: .97; 95% CI: .81–1.16)* **Smoking prevention (RR: .81; 95% CI: .70–.93)** *Smoking cessation (RR: .96; 95% CI: .90–1.02)*
	Policy interventions	**30-day smoking prevalence (−1.5% [95% CI: −6.0% to −2.9%])**
	Incentives	*Smoking uptake at longest follow-up (RR: 1.00; 95% CI: .84–1.19)*
	Multicomponent interventions	**Lifetime smoking (RR: .73; 95% CI: .64–.82)** *30-day smoking (RR: .79; 95% CI: .61–1.02)* **Regular smoking (RR: .59; 95% CI: .42–.83)** **Smoking cessation (RR: 1.55; 95% CI: 1.16–2.06)** **Smoking cessation (RR: 1.56; 95% CI: 1.21–2.01)**
Alcohol use	School-based interventions	**Alcohol consumption (quantity/week/month; SMD: .13; 95% CI: .07–.19)** **Frequency of drinking days (SMD: .07; 95% CI: .02–.13)** *Frequency of heavy drinking (SMD: .07; 95% CI: −.01 to .14)* *Alcohol-related problems (SMD: .06; 95% CI: −.03 to .15)* *Alcohol use (>13 months) (RR: .94; 95% CI: .85–1.04)* **Alcohol consumption (RR: .34; 95% CI: .11–.56)**
	Digital platforms	*Frequency of heavy drinking (<5 weeks; effect size: −.01; 95% CI: −.15 to .14)* *Alcohol-related problems (<5 weeks; effect size: .14; 95% CI: −.24 to .51)* *Frequency of heavy drinking (>6 weeks; effect size: −.07; 95% CI: −.27 to .13)* **Alcohol-related problems (>6 weeks; Effect size: .16; 95% CI: .03–.30)**
	Policy interventions	**Total alcohol consumption (low alcohol content movies vs. high; MD: −.65; 95% CI: −1.23 to −.07]** **Total alcohol consumption (Nonalcohol commercials vs. alcohol commercials; MD: −.73; 95% CI: −1.30 to −.16)** *Volume of alcohol sales (Total advertising ban vs. partial advertising ban; MD: −11.11; 95% CI: −27.56 to 5.34)*
Drug use	School-based interventions	*Marijuana use (<12 months; RR .79; 95% CI: .59–1.05)* **Marijuana use (>12 months; RR .83; 95% CI: .69–.99)** *Hard drug use (<12 months; RR .85; 95% CI: .63–1.14)* *Hard drug use (>12 months; RR .86; 95% CI: .39–1.9)* **Any drug use (<12 months; RR: .76; 95% CI: .64–.89)** *Cannabis use (RR: .58; 95% CI: .55–.62)*
Combined substance abuse	School-based interventions	**Alcohol frequency (brief intervention vs. assessment only; SMD −.91; 95% CI: −1.21 to −.61)** *Cannabis dependence (brief intervention vs. assessment only; SMD −.26; 95% CI: −.57 to .36)* *Alcohol frequency (brief intervention vs. information provision; SMD: −.01; 95% CI: −.20 to .18)* *Cannabis dependence (brief intervention vs. information provision; SMD: −.09; 95% CI: −.27 to .09)*
	Mentoring	*Alcohol use (SMD: −.09; 95% CI: −.32 to .14)* *Marijuana use (SMD: −.20; 95% CI: −.43 to .03)*
	Multicomponent intervention	**Alcohol and other drugs aggregate outcomes (RR: .24; 95% CI: .11–.37)** **Alcohol frequency outcomes (RR: .44; 95% CI: .12–.77)** **Alcohol quantity outcomes (RR: .05; 95% CI: .02–.08)** **Heavy/binge drinking (RR: .14; 95% CI: .05–.22)** *Marijuana use (RR: .22; 95% CI: −.09 to .52)*

Bold indicates significant impact. Italics indicates nonsignificant impact.

CI = confidence interval; RR = relative risk; SMD = standard mean difference.
